# Irisin hampers β-amyloid-induced microglial inflammation *via* the miR-451a/TLR4/NLRP3 axis

**DOI:** 10.3389/fimmu.2026.1761520

**Published:** 2026-04-23

**Authors:** Roberta Mancuso, Marina Saresella, Riccardo Nuzzi, Simone Agostini, Ivana Marventano, Ambra Hernis, Francesca La Rosa, Federica Piancone, Mario Clerici

**Affiliations:** 1 IRCCS Fondazione Don Carlo Gnocchi, Milan, Italy; 2 Department of Pathophysiology and Transplantation, University of Milan, Milan, Italy

**Keywords:** irisin, microglia, miR-451a, neuroinflammation, NLRP3, rehabilitation

## Abstract

**Introduction:**

Neuroinflammation, which is driven by microglial activation, contributes to neurodegeneration. The myokine irisin exerts anti-inflammatory effects, potentially through microRNA-mediated regulation of inflammasome components. However, the underlying molecular mechanisms remain incompletely defined. In this *in vitro* study we investigated whether irisin attenuates the β-amyloid (Aβ)-induced activation of human microglia via miR-451a-, miR-223-3p-, and miR-7-5p-dependent modulation of NLRP3-related genes.

**Methods:**

For this aim, human immortalized microglia (hTERT) were LPS primed and Aβ_1-42_, stimulated in the presence or absence of irisin. The expression of NLRP3, TLR4, caspase-1, IL-1β, IL-18, PYCARD, and selected microRNAs (miR-451a, miR-223-3p, and miR-7-5p) was quantified by digital droplet PCR. TLR4 expression in hTERT cells was analyzed by flow cytometry and intracellular ASC speck formation with NLRP3 colocalization and NF-κB nuclear translocation was measured by imaging flow cytometry. Cytokine release was measured in the supernatants using ELISA. Loss of function assays were performed in hTERT cells transfected with either the miR-451a inhibitor or scrambled molecules.

**Results and discussion:**

Irisin treatment was associated with reduced Aβ_1-42_-induced microglial activation, as showed by decreased TLR4 expression, reduced markers of NLRP3 inflammasome activation (including ASC-speck formation, gene and protein expression), and NF-kB nuclear translocation. This effect was accompanied by the upregulation of miR-451a and miR-7-5p and the downregulation of miR-223-3p expression. miR-451a inhibition attenuated the irisin-associated anti-inflammatory effects, suggesting a contributory role for miR-451a in the modulation of TLR4/NLRP3 pathway in this *in vitro* model. To summarize, this work showed that, in an *in vitro* model of Aβ_1-42_-activated human microglia, irisin is associated with modulation of microRNA expression and NLRP3 inflammasome-related pathways. These results provide mechanistic insight into potential interaction between exercise-related factors and microglial inflammatory responses; their possible relevance to *in vivo* conditions and disease contexts will nevertheless clearly requires further investigation.

## Introduction

Microglia are the primary resident immune cells in the central nervous system (CNS) essential for tissue surveillance, response to pathogens or injury and misfolded protein accumulation (1). In Alzheimer’s disease (AD), microglial cells contribute to the uptake and clearance of amyloid-beta (Aβ) and Tau protein aggregates, but their chronic activation leads to sustained neuroinflammation and consequent neurodegeneration (2, 3). Aβ aggregates and hyperphosphorylated Tau proteins can over-activate the Nod-like receptor pyrin domain containing 3 (NLRP3) inflammasome in microglia and in peripheral monocytes (4–6), driving the chronic secretion of pro-inflammatory cytokines such as IL-1β and IL-18, thereby contributing to cognitive decline (7).

The NLRP3 inflammasome is a multiprotein complex that requires a two-step activation process: a “priming” signal, via lipopolysaccharide (LPS)-toll like receptor 4 (TLR4) and NF-κB-dependent transcription of NLRP3, pro-IL-1β, pro-IL-18, and pro-caspase-1; a following “activation” signal that promotes the NLRP3 inflammasome complex assembly and the secretion of IL-1β and IL-18 (8). In experimental settings, the combination of LPS priming and Aβ stimulation is widely used to induce robust and acute inflammasome response in microglia and, although it clearly does not approach the complexity of AD pathogenesis, it can be interpreted as a model of inflammatory priming relevant to specific aspects of Aβ-associated microglia activation.

Various components of the NLRP3 inflammasome pathway have been investigated in recent years as potential therapeutic targets for AD, to reduce neurodegeneration, Aβ accumulation and to limit inflammation (7, 9–11). Notably, recent results have also suggested a neuroprotective role of irisin in AD (12, 13).

Irisin, a myokine derived from the proteolytic cleavage of fibronectin type III domain-containing 5 (FNDC5) transmembrane protein during physical exercise, exerts neuroprotective, anti-inflammatory and antioxidant effects in both peripheral tissues (i.e. adipose tissue, muscle, liver) and the CNS, as it can cross the blood-brain barrier (14). Given the association between irisin release and physical activity, rehabilitation approaches and exercise-based interventions have been proposed as potential strategies to modulate the progression of AD (15). Notably, irisin was recently suggested to suppress NLRP3 activation (16), promotes neuronal survival and synaptic plasticity, induces brain-derived neurotrophic factor (BDNF) expression, improves mitochondrial function (14), and decreases Aβ deposition and Tau phosphorylation, ultimately improving cognitive performance (17, 18). Through binding to microglial integrin αVβ5, irisin influences several pathways including AMPK, TLR4/MyD88, AKT and MAPK1/3, as well as NF-kB (14), thereby suppressing pro-inflammatory cytokine production and reactive oxygen species generation (19).

MicroRNAs (miRNAs) are small noncoding RNAs that regulate gene expression post-transcriptionally (20). Different miRNAs have been implicated in mediating the anti-inflammatory effects of irisin in various tissues (21–24). The possible interaction between irisin, miRNAs and suppression of inflammation in AD is still unclear.

In this study, we used a human microglial cell model primed with LPS and stimulated with Aβ as an experimental paradigm to investigate acute inflammatory activation and inflammasome signaling relevant to Aβ-associated microglial responses.

We investigated whether irisin modulates three microRNAs -miR-451a, miR-223-3p, and miR-7-5p –which were selected based on prior evidence linking them to NLRP3 inflammasome (25–27). We aimed at shedding some light on the miRNA-mediated mechanisms associated with the anti-inflammatory effect of irisin. Results herein provide novel insight into molecular pathways that may play a role in the pathogenesis of AD-associated neuroinflammation.

## Materials and methods

### Cell culture and treatments

Immortalized human microglial hTERT cells (Applied Biological Materials Inc., Richmond, BC, Canada) were cultured at 37 °C with 5% CO_2,_ in PriGrow III medium (Applied Biological Materials Inc.) supplemented with 10% heat-inactivated fetal bovine serum (FBS; PAN-Biotech; Aidenbach, Germany) and 1% penicillin/streptomycin. Cells were seeded into 24-well plates at 1 × 10^6^ cells/well and cultured in medium alone, or primed for 2 h with LPS (1 μg/mL) and then 2.5 µM Aβ_1-42_ (Phoenix Pharmaceuticals, Burlingame, CA, US) in presence/absence of irisin (100 ng/mL; R&D Systems, Minneapolis, MN, US; Catalog No. 8880-IR) for 1 or 22 hours at 37 °C with 5% CO_2_. The experimental groups included: 1) untreated control (med), 2) LPS + Aβ_1-42_ (positive control), and 3) LPS + Aβ_1-42_ + irisin. Each condition was tested in triplicate. After 1 h of treatment, cells were harvested for the extraction of total RNA (containing both mRNA and miRNA) to analyze gene and miRNA expression. After 22 h of incubation, hTERT cells were harvested by adding 60 μL/well of trypsin-EDTA solution (Sigma-Aldrich, St. Louis, MO, US) for 5 min at 37 °C) and centrifuged for 10 min at 1500×*g*. Cells were then used for flow cytometry (TLR4 expression) and for imaging flow-sight (apoptosis-associated speck-like protein containing a CARD (ASC) speck/NLRP3 colocalization and NF-kB nuclear translocation). The supernatants were frozen at -20 °C for cytokine detection.

### Cell viability and cytotoxicity assays

Cell viability was evaluated by the Trypan blue exclusion assay, as previously reported (28), using a TC20 Automatic Cell Counter (Bio-Rad, Hercules, CA, US). Cell survival was expressed as a percentage of untreated cells. Cytotoxicity was measured by quantifying lactate dehydrogenase (LDH) release in cell culture supernatants using an LDH cytotoxicity detection kit (Sigma-Aldrich) following the manufacturer’s instructions. The results obtained after 1 and 24 h of culture showed that cell viability was > 85% under all incubation conditions.

### Cytokines production

Supernatants were thawed and centrifuged to eliminate debris. Active caspase-1 (p20), IL1-β, and IL-18 concentrations were measured using sandwich immunoassays according to the manufacturer’s recommendations (Quantikine Immunoassay; R&D Systems, or Thermo Fisher Scientific, Waltham, MA, USA). A plate reader (Sunrise, Tecan, Mannedorf, Switzerland) was used to determine the optical density (OD) at 450/620 nm. All samples were analyzed in duplicate. The sensitivity was as follows: IL-1β: 1 pg/mL; IL-18: 5.15 pg/mL; caspase1:1.24 pg/mL. Assay Range was as follows: IL-1β: 3.9 -250 pg/mL; IL-18: 15.6 -1000 pg/mL; caspase-1: 6.3 -400 pg/mL.

### Immunofluorescence staining

hTERT cells harvested after being incubated in different culture conditions were used as follows:

1) to analyze surface expression of TLR4, the hTERT cells were incubated for 1 hour at 4 °C with 5 μL of PC7-anti human TLR4 (clone UT41, isotype Mouse IgG1k) (eBioscience, San Diego, CA, US) at room temperature (RT) in the dark, followed by washing with phosphate-buffered saline (PBS) by centrifugation at 1,500 × g for 10 min at 4 °C and resuspension in 100 μL of 1% paraformaldehyde (PFA) in PBS for 15 min. The cells were then washed, resuspended in 500 μL ice-cold PBS, and analyzed by Flow Cytometry.

2) To analyze inflammasome assembly (activation), hTERT cells were permeabilized with 100 μL of 0.1% saponin in PBS (Life Science VWR, Lutterworth, Leicestershire, UK) and incubated for 1 h at 4 °C in the dark with 5 μL of Alexa Fluor^®^ 488-anti human NLRP3 (Clone 768319, isotype RatIgG2a) (R&D Systems) and 5 μL of PE-anti human ASC (clone HASC-71, isotype mouse IgG1, Biolegend, San Diego, CA, US). After incubation, the cells were washed and fixed with 100 μL of PFA 1% in PBS for 15 min. The cells were then washed and resuspended in 500 μL ice-cold PBS for imaging flow-sight (AMNIS) analyzes.

3) to analyze NF-kB nuclear translocation, the AMNIS^®^ NF-kB translocation kit was used according to the manufacturer’s recommendations (Merck Kgaa, Darmstadt, Germany). Briefly, cells were fixed, permeabilized, and stained with anti human NF-κB (p50) Alexa Fluor^®^ 488 for 30 min at RT. After incubation, the cells were washed, fixed and 10μL of 7 AAD was added. After washing and centrifugation, the cells were resuspended in 500 μL ice-cold PBS for AMNIS analysis.

### Immunofluorescence analysis by flow cytometry and Imaging FlowSight

TLR4 expression analysis was performed using a Beckman-Coulter DxFlex flow cytometer equipped with three active lasers (405 nm, 488 nm, and 638 nm), 13 channels for fluorescence detection, and interfaced with Kaluza analysis software. Flow cytometry was performed using the fluorescence minus one (FMO) approach. Twenty thousand events were acquired and gated forward (FSC) and side scatter (SSC) properties were used to identify microglial hTERT cells.

For imaging-based analyzes, 2,000 events per sample were acquired, using a Flow-Sight imaging flow cytometer (Luminex, Austin, TX, US). ASC speck formation and NLRP3 colocalization were quantitatively analyzed using IDEAS analysis software 6.2 (Cytek Biosciences, Fremont, CA, USA) through internalization features, based on predefined masks. These included a whole cell mask defined by the bright field (BF) image and an internal mask defined by eroding the whole cell mask, allowing differentiation between diffuse or spot (speck) fluorescence pattern inside the cells. A threshold mask was used to separate all ASC-positive cell populations between ASC speck spot cells and ASC-diffuse cells according to the different diameters of the spot area. In the ASC speck, the spot shows a small area and a high maximum pixel intensity, and the opposite occurs in the ASC diffuse cells. NLRP3 expression was analyzed by internalization features utilizing a mask representing the whole cell, defined by the brightfield image, and an internal mask defined by eroding the whole cell mask.

Analysis of NF-kB translocation was performed by Nuclear Localization Wizard using Similarity Features. Briefly, nuclear translocation occurs when the activated form of NF-κB translocates into the nucleus and its fluorescence signal overlaps with the nuclear fluorescence signal (7AAD). Bright detail similarity is specifically designed to compare the small bright details of two images and can be used to identify and quantify the colocalization of the two probes (NF-kB and 7AAD) in a defined region of interest. The similarity score is the log-transformed Pearson correlation coefficient, and is a measure of the degree to which two images are linearly correlated within a masked region, calculated over a double-positive region (NF-kB+7AAD+). Only single, in-focus cells were included in the analysis based on standard gating strategies. All acquisition and analysis parameters were applied uniformly across experimental conditions. Image analysis was performed in a blinded manner with respect to experimental groups to minimize operator bias.

### RNA extraction and gene expression analysis by ddPCR

Total RNA, including both miRNA and mRNA, was extracted from each well after 1-hour incubation under different conditions using the “miRNeasy tissue/cells advanced Micro kit” (Qiagen, Hilden, Germany) on a semi-automated Qiacube workstation (Qiagen) according to the manufacturer’s protocol. RNA and miRNA concentrations were quantified separately using a Qubit 2.0 fluorometer with the Qubit^®^ RNA HS Assay and Qubit^®^ miRNA Assay kits (High Sensitivity, ThermoFisher, Foster City, CA, US) respectively.

The expression of NLRP3 inflammasome-related genes (NLRP3, IL-1β, caspase 1, IL-18, and ASC-coding gene PYCARD), and TLR2 and TLR4, was measured using a one-step multiplex RT-ddPCR assay with target-specific primers and fluorescent probes, as previously described (10). **Supplementary Table 1** shows the assays ID and probe fluorophore for each target gene. Briefly, 5 µL of RNA (diluted 1:100) was added to a 20 μL reaction mix containing One-Step RT-ddPCR Supermix (Bio-Rad), reverse transcriptase, dithiothreitol (DTT), target-specific primer/probes assay, and nuclease-free water. Droplet generation and PCR amplification were performed under standard conditions. Droplets were read with a QX200 Droplet Reader (Bio-Rad), and data were analyzed using the QuantaSoft software (v1.7.4.0917, Bio-Rad). A sample was considered positive if ≥ 3 droplets showed fluorescence. The results were normalized to the amount of input RNA and expressed as copies/ng RNA.

### miRNAs detection by ddPCR

Following reverse transcription (obtained from 4 µL RNA input) with the miRCURY LNA RT Kit (Qiagen), the expression of miR-223-3p, miR-7-5p, and miR-451a was analyzed by ddPCR with LNA™-specific primers (Qiagen) on the QX200 ddPCR system (Bio-Rad). The experimental workflow and data analyzes have been previously described (10). **Supplementary Table 1** shows the assays ID for each miRNA. Briefly, 3 μL of diluted cDNA (1:100) was mixed with ddPCR EvaGreen Supermix (Bio-Rad) and LNA™-specific primers. No-template and RT-negative controls were included in each run to monitor for non-specific amplification. The number of copies per well, calculated using QuantaSoft software (v1.7.4.0917, Bio-Rad), was normalized to the input RNA concentration and expressed as copies/ng.

### miRNAs transfection

To investigate the role of mir-451a, hTERT cells were transfected with either an miR-451a inhibitor (Cohesion Biosciences, London UK, cat CIH0221) or a scrambled miRNA negative control (cat. CIH0000) using the ScreenFect^®^ Aplus Transfection Reagent (ScreenFect, Eggenstein-Leopoldshafen, Germany) following the manufacturer’s one-step protocol, which allows simultaneous plating and transfection of cells. The miR-451a inhibitor or scrambled miRNA was precomplexed with ScreenFect Aplus reagent in the supplied dilution buffer and the mixture was added directly to the well plate. After preliminary experiments to select the appropriate concentration, hTERT cells were transfected overnight with a final concentration of 50nM for inhibitor and scramble. After transfection, cells were exposed to the same conditions as previously described: 1) untreated control (med), 2) LPS + Aβ_1-42_, and 3) LPS + Aβ_1-42_ + irisin. One hour later, cells were harvested for RNA/miRNA quantification, and after 22 h of incubation for imaging FlowSight (ASC speck and NLRP3 colocalization and NF-kB translocation) and flow cytometry (TLR4), the supernatants were frozen at -20 °C for cytokine analysis (see above).

### Statistical analysis

The experiments were independently repeated three times. For each experiment, each condition was measured in triplicate. The mean value of technical replicate was used for the statistical analysis. Parametric data are expressed as mean ± standard deviation (SD), while non-parametric data are expressed as median and interquartile range (IQR). For normally distributed data, one-way ANOVA was used to compare three groups and significant differences were analyzed using the Tukey’s *post-hoc* test for pairwise comparisons. For non-parametric data, the Kruskal–Wallis test was used, followed by pairwise Mann–Whitney U test with Bonferroni correction for multiple comparisons. Statistical significance was set at p ≤0.05. Data were analyzed using MedCalc^®^ software (version 14.10.2, Ostend, Belgium).

## Results

### Irisin downregulates TLR4 expression and density in LPS+Aβ_1–42_ stimulated cells

**Figure 1:**
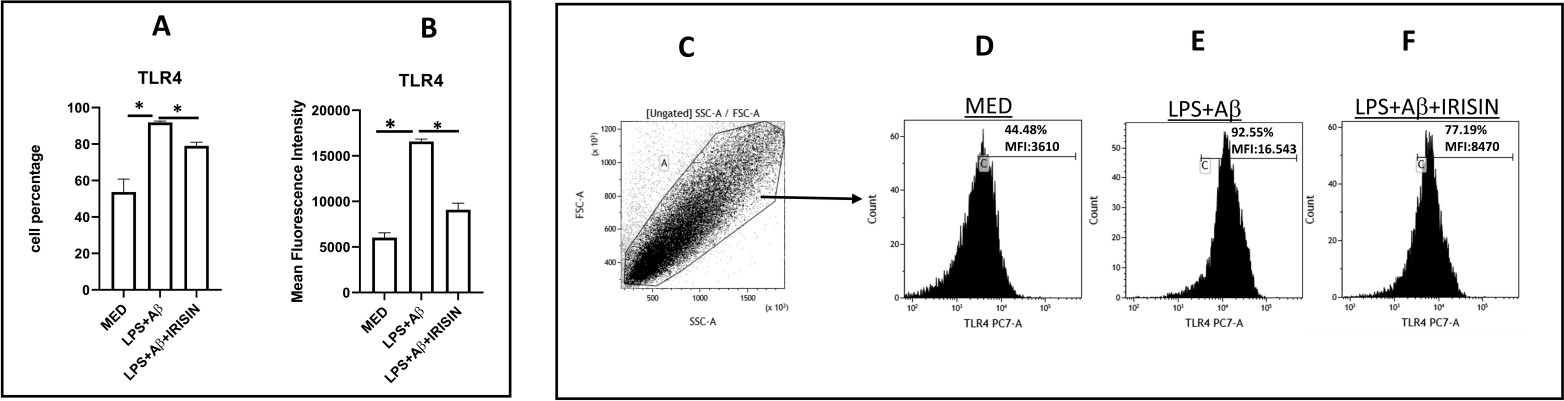
Summary of the results of TLR4 expressing cell percentage **(A)** and surface density (mean fluorescence intensity, MFI) **(B)** in LPS-primed and Aβ_1-42_-stimulated hTERT microglia in presence or absence of irisin. The MFI was calculated based on TLR4-expressing hTERT percentage. Data are presented as median and interquartile range from n=3 independent experiments. Statistical significance is indicated: * p<0.05. A representative dot plot of forward (FS) and side scatter (SS) **(C)** gating hTERT microglia (Gate A) and histograms representing TLR4-expressing hTERT microglia analyzed by flow-cytometry in unstimulated (MED) **(D)**, LPS-primed Aβ_1-42_-stimulated hTERT microglia in absence **(E)** or presence **(F)** of irisin are shown. In the upper right corner of histograms, the percentage and MFI of TLR4 are shown.

Initial analyzes verified that irisin can modulate TRL4 expression. The results showed this to be the case, as both the percentage of TLR4 expressing-cells (79%) (**Figure 1A**) and TLR4 density on the cell surface (MFI) (**Figure 1B**) were significantly reduced in hTERT cells that were LPS-primed and Aβ_1–42_ stimulated in the presence of irisin, compared to the results obtained in the absence of irisin (p=0.01 and p=0.009, respectively).

### Irisin downregulates NF-κB nuclear translocation in LPS+Aβ_1–42_ stimulated cells

**Figure 2:**
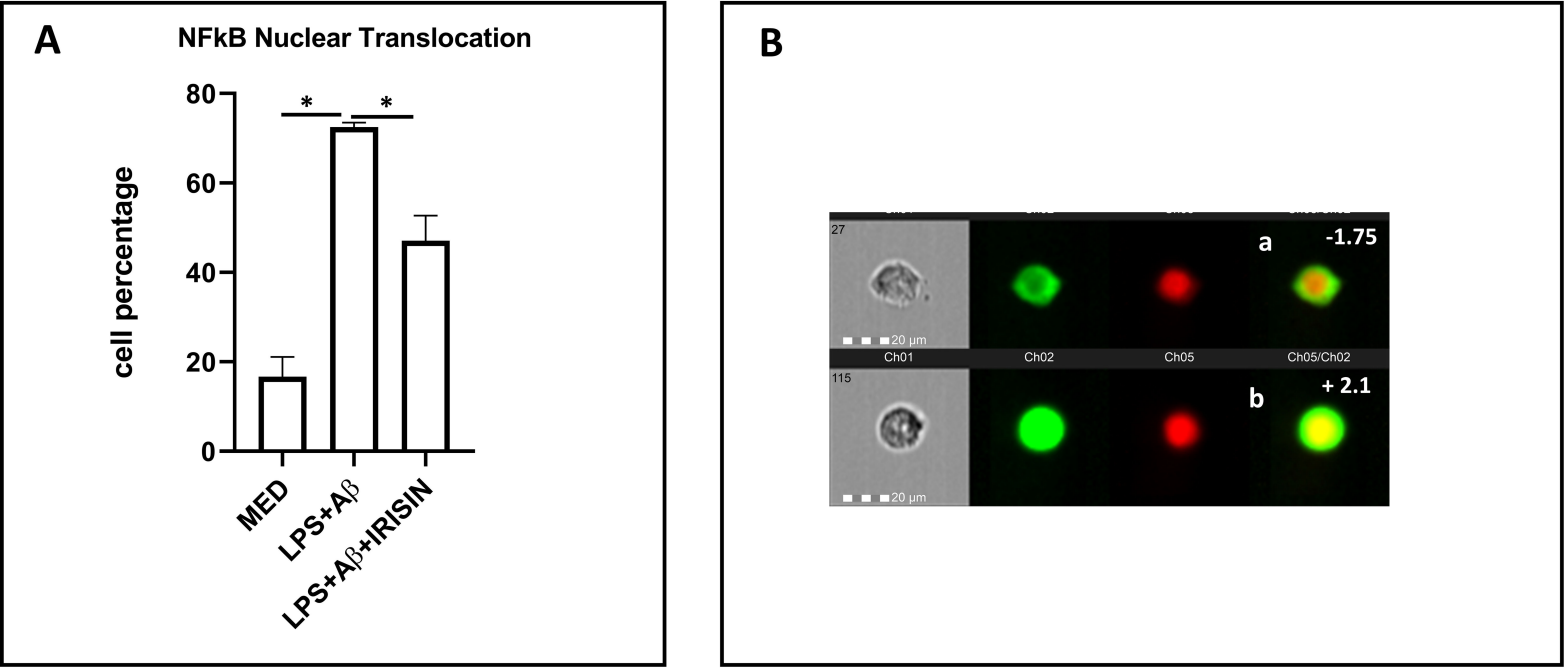
**(A)** Summary of the results of cell percentage expressing NF-kB nuclear translocation in unstimulated (MED) or LPS-primed and Aβ_1-42_-stimulated hTERT microglia in presence or absence of irisin. Data are presented as median and interquartile range from n=3 independent experiments. Statistical significance is indicated: *p<0.05. **(B)** Representative images from FlowSight (AMNIS) analysis of hTERT microglia expressing NF-kB protein and nuclear DNA. First column: brightfield (BF); second column: NF-kB-FITC fluorescence; third column: 7AAD fluorescence (DNA stain); fourth column: NF-kB/7AAD merged fluorescence. NF-kB nuclear translocation is indicated by colocalization with nuclear DNA: **(a)** microglia with nuclear 7AAD staining (red) and cytoplasmatic NF-kB staining (green), indicating lack of nuclear translocation (similarity score: − 1.75); **(b)** colocalization of NF-kB and DNA (yellow) indicating nuclear translocation (similarity score: + 2.1). The similarity score is calculated to verify protein colocalization.

Nuclear translocation of NF-κB is modulated by irisin. Thus, the percentage of hTERT cells showing nuclear translocation of NF-κB upon LPS priming and Aβ_1–42_ stimulation was significantly reduced when stimulation was done in the presence of irisin (p=0.012) (**Figure 2A**).

### Irisin downregulates NLRP3 inflammasome activation in LPS+Aβ_1–42_ stimulated cells

**Figure 3:**
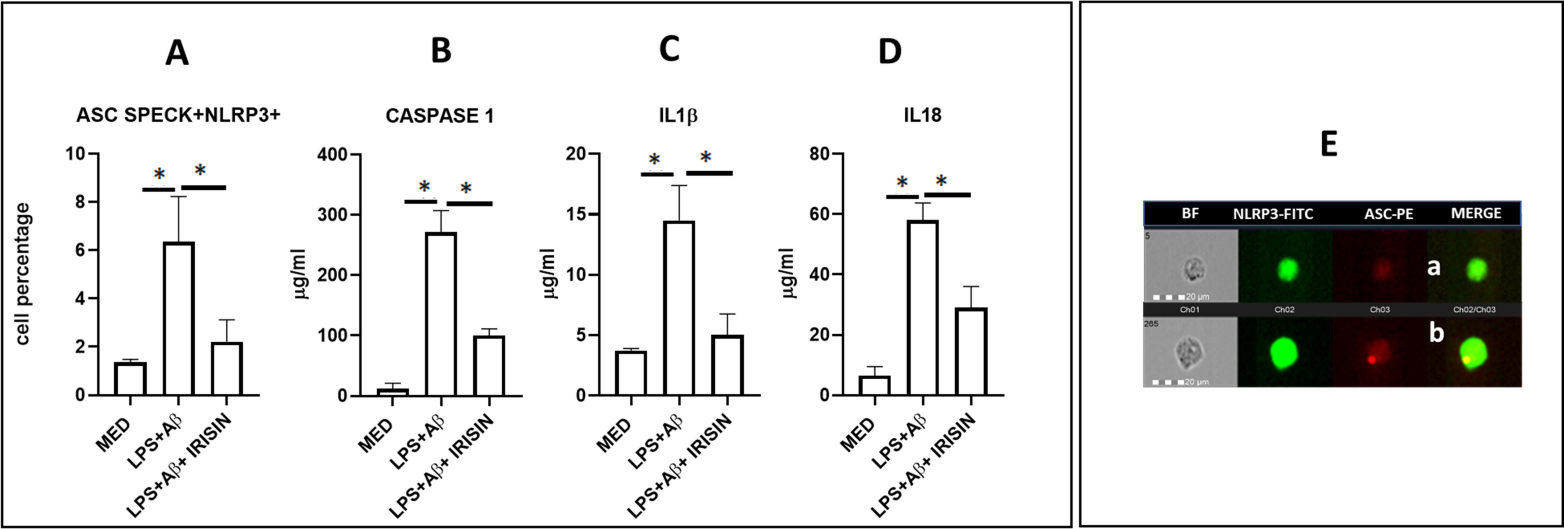
Intracellular ASC speck formation with NLRP3 colocalization was analyzed by Flow-Sight (AMNIS), and activated caspase-1(p20), IL-1β and IL-18, were detected in the supernatants by ELISA. Summary results of the cell percentage expressing intracellular NLRP3/ASC speck colocalization **(A)**, activated-caspase 1 (p20) release **(B)**, IL-1β **(C)** and IL-18 **(D)** production in the supernatants of unstimulated (MED) or LPS-primed and Aβ_1-42_-stimulated hTERT microglia in presence or absence of irisin are shown. Data are presented as median and interquartile range from n=3 independent experiments. Statistical significance is indicated: *p<0.05. **(E)** Representative images of hTERT microglia expressing intracellular NLRP3 and ASC protein. First column: microglia in brightfield (BF); second column: NLRP3-FITC-labeled microglia; third column: ASC-PE- labeled microglia; fourth column: ASC-PE-labeled cells merged with NLRP3-FITC fluorescence-labeled microglia. **(a)** cell with diffuse ASC fluorescence (red) and lack of colocalization with NLRP3 (green). **(b)** ASC speck formation colocalized with NLRP3 (yellow).

Intracellular NLRP3 colocalization with ASC speck induces caspase-1 activation, resulting in the production of IL1-β and IL-18. The results showed that the percentage of LPS-primed and Aβ_1–42_ stimulated- hTERT cells in which intracellular NLRP3 colocalized with ASC speck was significantly reduced by irisin (p=0.001) (**Figure 3A**).

The concentrations of activated caspase-1 (p20), IL-1β, and IL-18 were significantly reduced in the supernatants of LPS-primed and Aβ_1–42_ stimulated-hTERT cells in the presence of irisin (p=0.012, p=0.03 and p=0.018, respectively). These results are shown in **Figures 3B–D**.

### Irisin reduces TLR4 but not TLR2 gene expression in LPS+Aβ_1–42_ stimulated cells

**Figure 4:**
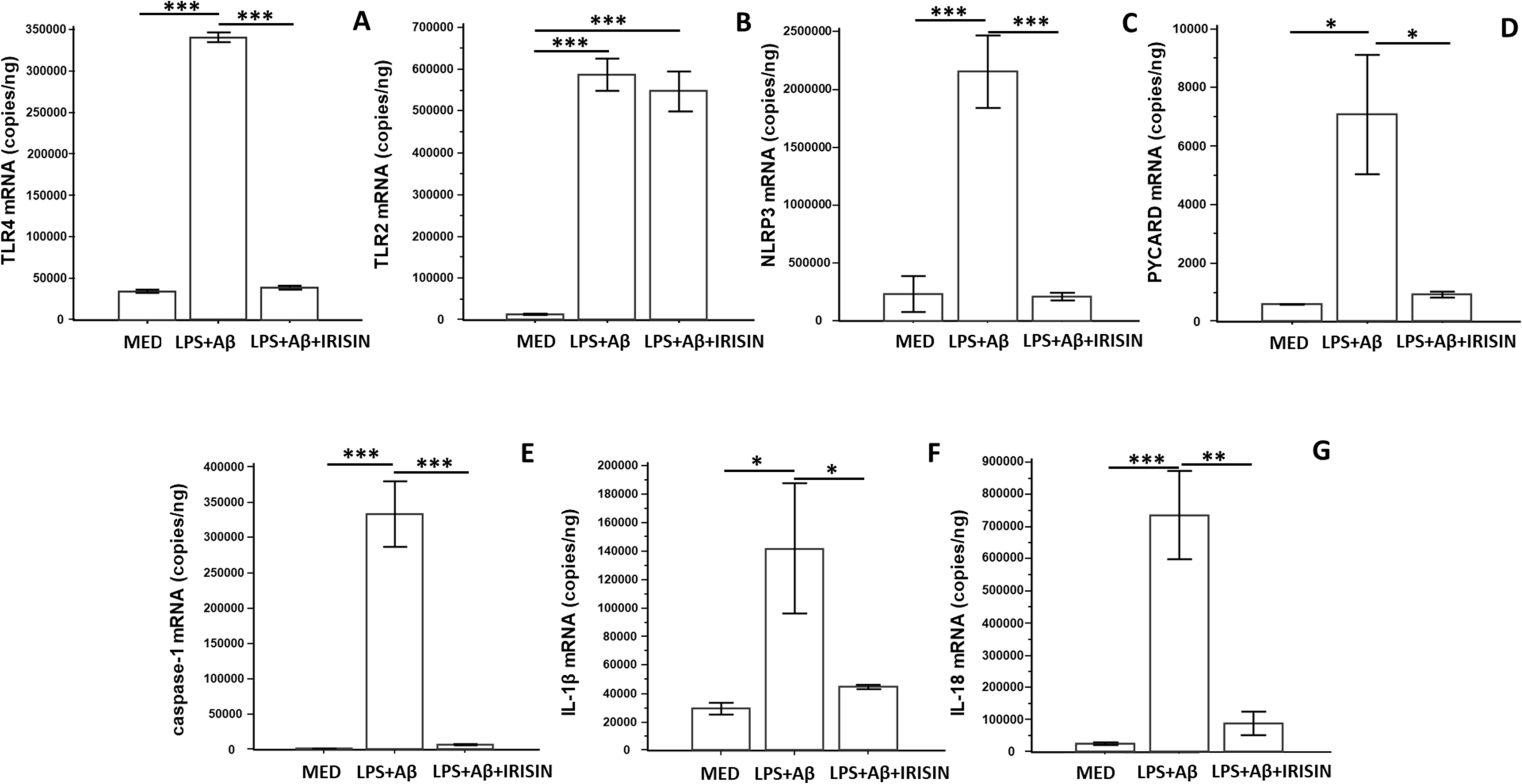
hTERT microglia were unstimulated (MED) or LPS-primed and Aβ _1-42-_stimulated in presence or absence of irisin. mRNA expression of NLRP3 inflammasome components (**A**: TLR4; **B**: TLR2, **C**: NLRP3, **D**: PYCARD; **E**: caspase-1 **F**: IL-1β; **G**: IL-18) was analyzed by ddPCR. Data are expressed as copies/ng of RNA and presented as mean ± SD from n=3 independent experiments. Statistical significance is indicated: *p<0.05; **p ≤ 0.001; ***p ≤ 0.0001.

TLR4 and TLR2 gene expression was analyzed under all experimental conditions. As expected, LPS-primed and Aβ_1–42_ stimulation resulted in significant upregulation of TLR4 and TLR2 mRNA compared to cells cultured in the medium alone (TLR4: p<0.0001; TLR2: p<0.0001) (**Figures 4A, B**). The presence of irisin in the culture medium significantly downregulated TLR4 expression (p <0.0001), whereas TLR2 expression remained unchanged.

### Irisin downregulates NLRP3-related genes expression in LPS+Aβ_1–42_ stimulated cells

To determine whether the inhibitory effects of irisin on the NLRP3 inflammasome are associated with the transcriptional suppression of NLRP3-related genes, the mRNA expression of NLRP3, IL-1β, caspase-1, PYCARD, and IL-18 was assessed by ddPCR. The results showed that the transcription of these genes was strongly suppressed when hTERT cells were LPS-primed and Aβ_1–42_ was stimulated in the presence of irisin (p<0.05) (**Figures 4C–G**).

Notably, incubation of hTERT cells with irisin alone did not induce any significant changes in the expression of these genes (**Supplementary Figure 1**).

### Effect of irisin on miR-451a, miR-7-5p and miR-223-3p expression in LPS+Aβ_1–42_ stimulated cells

**Figure 5:**
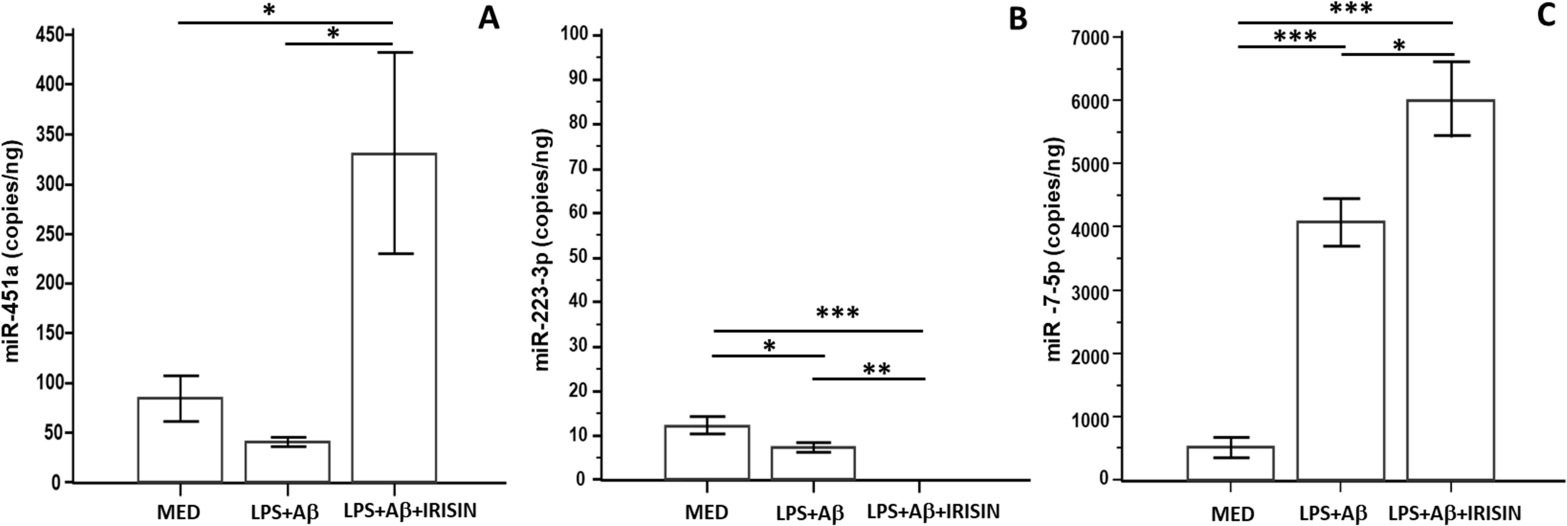
hTERT microglia were unstimulated (MED) or LPS-primed and Aβ_1_-_42_-stimulated in presence or absence of irisin. Expression of miR-451a **(A)**, miR-223-3p **(B)** and miR-7-5p **(C)** was analyzed by ddPCR. Data are expressed as copies/ng and presented as mean ± SD from n=3 independent experiments. Statistical significance is indicated: *p<0.05; **p ≤ 0.001; ***p<0.0001.

miR-451a, miR-223-3p, and miR-7-5p were selected based on their reported ability to regulate the NLRP3 inflammasome activation (25–27). The effect of irisin on the expression of these miRNAs was analyzed using ddPCR in LPS-primed and Aβ_1-42_ -activated cells in the presence or absence of irisin. The results showed that miR-451a expression was decreased in LPS-primed and Aβ_42_-activated cells and significantly increased in the presence of irisin (p=0.0059 *vs.* untreated, p=0.0026 *vs.* LPS+ Aβ_1-42_) (**Figure 5A**).

miR-223-3p expression was significantly reduced in LPS-primed and Aβ_42_-activated cells (p=0.0075*)*. However, in contrast to previous results, irisin resulted in an even more significant reduction in miR-223-3p expression (p=0.0009 *vs.* LPS+ Aβ_1-42_) (**Figure 5B**).

In contrast, miR-7-5p expression was significantly increased by LPS+ Aβ_1–42_ stimulation (p=0.0001 *vs.* untreated) and further upregulated following the addition of irisin (p<0.0001 *vs.* untreated) (**Figure 5C**).

### Effect of miR-451a inhibition on TLR4 and NLRP3-related genes expression in LPS+Aβ_1–42_ stimulated cells

**Figure 6:**
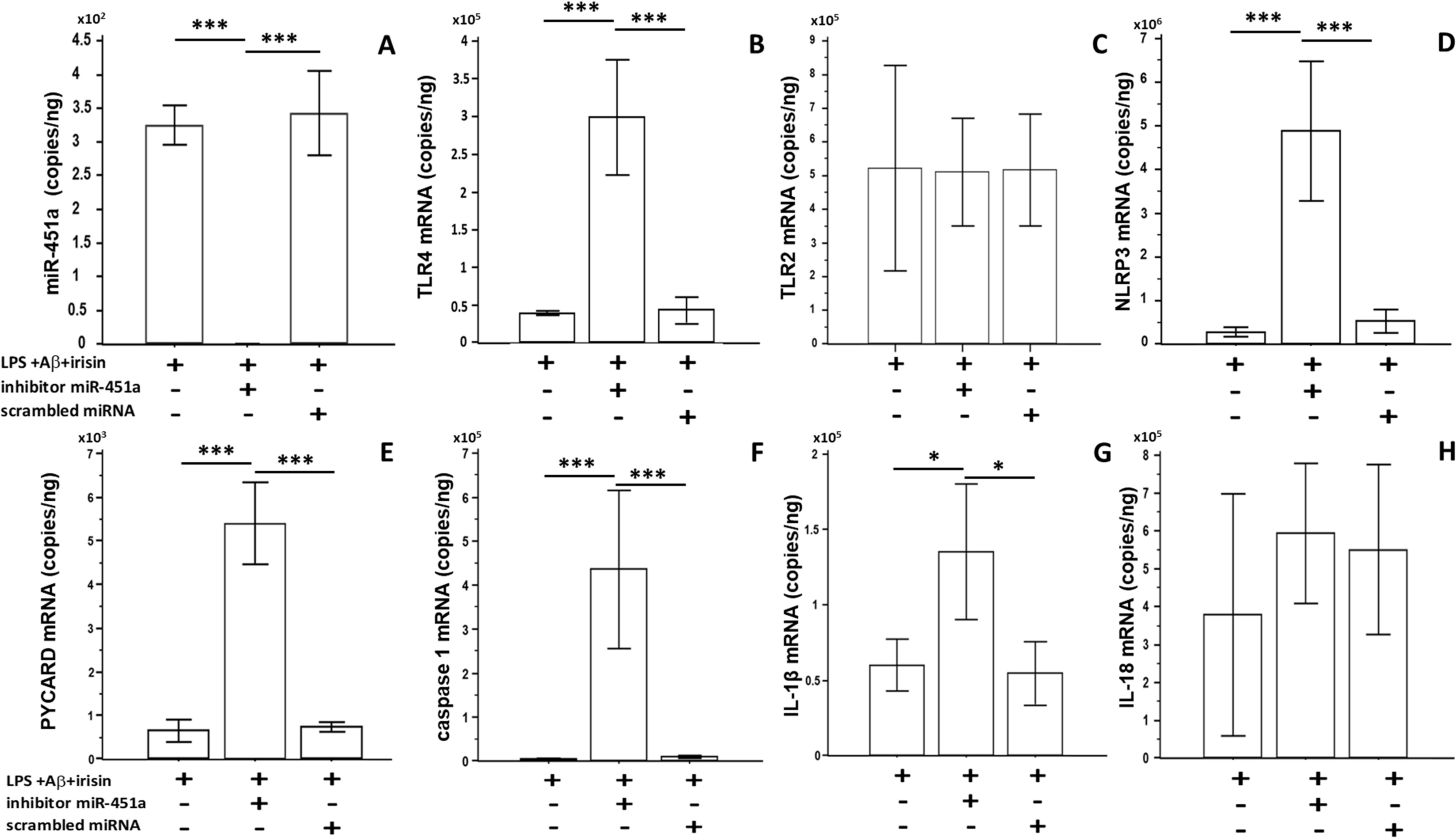
hTERT microglia were transfected overnight with miR-451a inhibitor or scrambled miRNA; then cells were LPS-primed and Aβ_1-42_-stimulated in presence or absence of irisin. Expression of miR-451a **(A)** and NLRP3 related genes **(B**: TLR4; **C**: TLR2; **D**: NLRP3; **E**: PYCARD; **F**: caspase-1; **G**: IL-1b; **H**: IL-18**)** was analyzed by ddPCR. Data are expressed as copies/ng and presented as mean ± SD from n=3 independent experiments. Statistical significance is indicated: *p<0.05; ***p<0.0001.

To investigate the functional role of miR-451a in TLR4 regulation and downstream NLRP3 inflammasome activation, hTERT microglial cells were transfected with a miR-451 inhibitor to suppress its expression. hTERT cells transfected with scrambled miRNA were used as negative controls. Following transfection, cells were cultured alone or primed with LPS and Aβ_1-42_-stimulated in the presence or absence of irisin. Results showed that transfection with anti-miR-451a, but not with the scrambled negative control, completely suppressed miR451a expression (**Figure 6A**).

Analysis of TLR4, NLRP3, caspase-1, IL-1β, and PYCARD gene expression in cells transfected with the miR-451a inhibitor showed that the suppressive effect of irisin on the transcription of these genes was reversed by miR-451a silencing. The effect of irisin on the transcription rate of these genes is likely to be mediated by this miRNA.

In contrast, IL-18 and TRL2 expression was not modified by miR-451a silencing, indicating that the expression of these genes is modulated by other molecular pathways. The results are presented in **Figures 6B–H**.

### Effect of miR-451a inhibition on TLR4 expression and NF-kB nuclear translocation in LPS+Aβ_1–42_ stimulated cells

**Figure 7:**
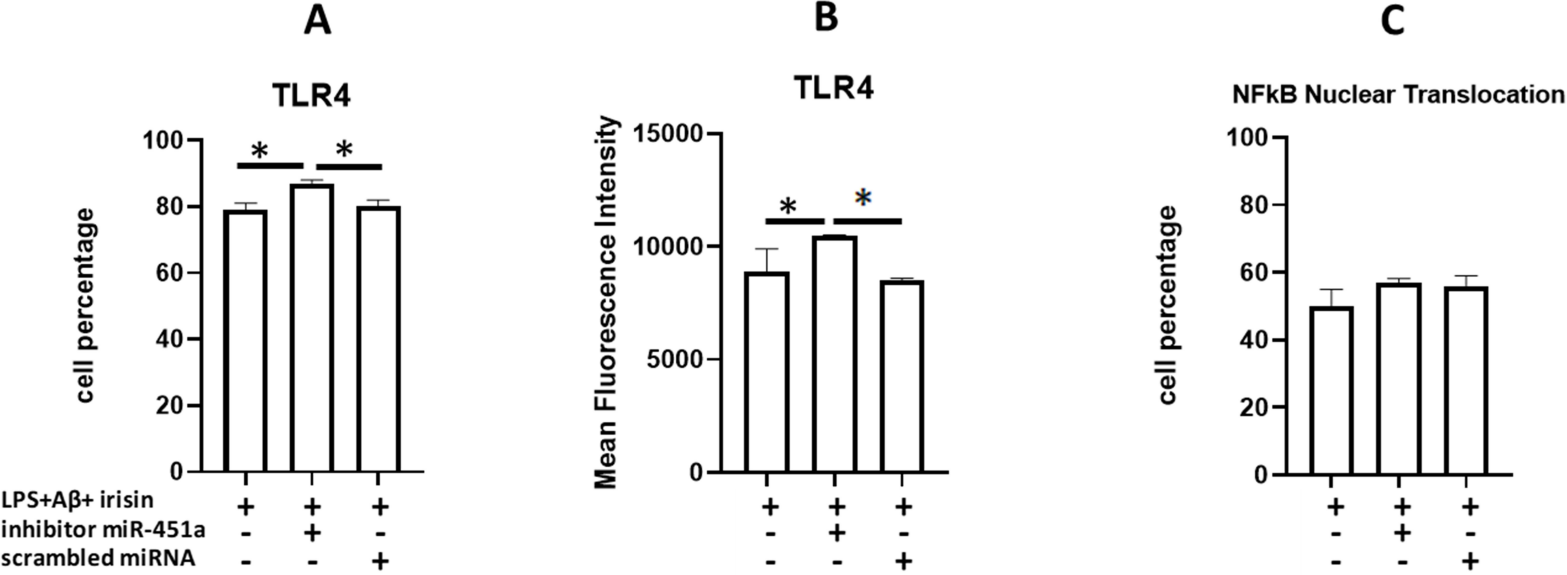
Summary of TLR4-expressing cells percentage **(A)**, TLR4 surface density (MFI) **(B)** and NF-kB nuclear translocation percentage **(C)** in untransfected (MED) or transfected with miR-451a inhibitor or scrambled miRNA hTERT microglia. Cells were LPS-primed and Aβ_1-42_- stimulated in presence of irisin. MFI values were calculated based on the percentage of TLR4-expressing microglia. Data are presented as median and interquartile range from n=3 independent experiments. Statistical significance is indicated: *p<0.05.

Silencing of miR-451a expression resulted in significant upregulation of both the percentage of TLR4 expressing cells (87%) (**Figure 7A**) and the density of the receptor (MFI) on the cell surface (**Figure 7B**) compared to untransfected hTERT cells (p=0.02 and p=0.03 respectively) and hTERT cells transfected with the scrambled peptide (p=0.02 and p=0.03, respectively).

The inhibitory effect of irisin on NF-kB nuclear translocation was hampered by miR-451a silencing, although this result was not statistically significant (compared with cells transfected with the scrambled peptide) (**Figure 7C**).

### Effect of miR-451a inhibition on NLRP3 inflammasome modulation in LPS+Aβ_1–42_ stimulated cells

**Figure 8:**
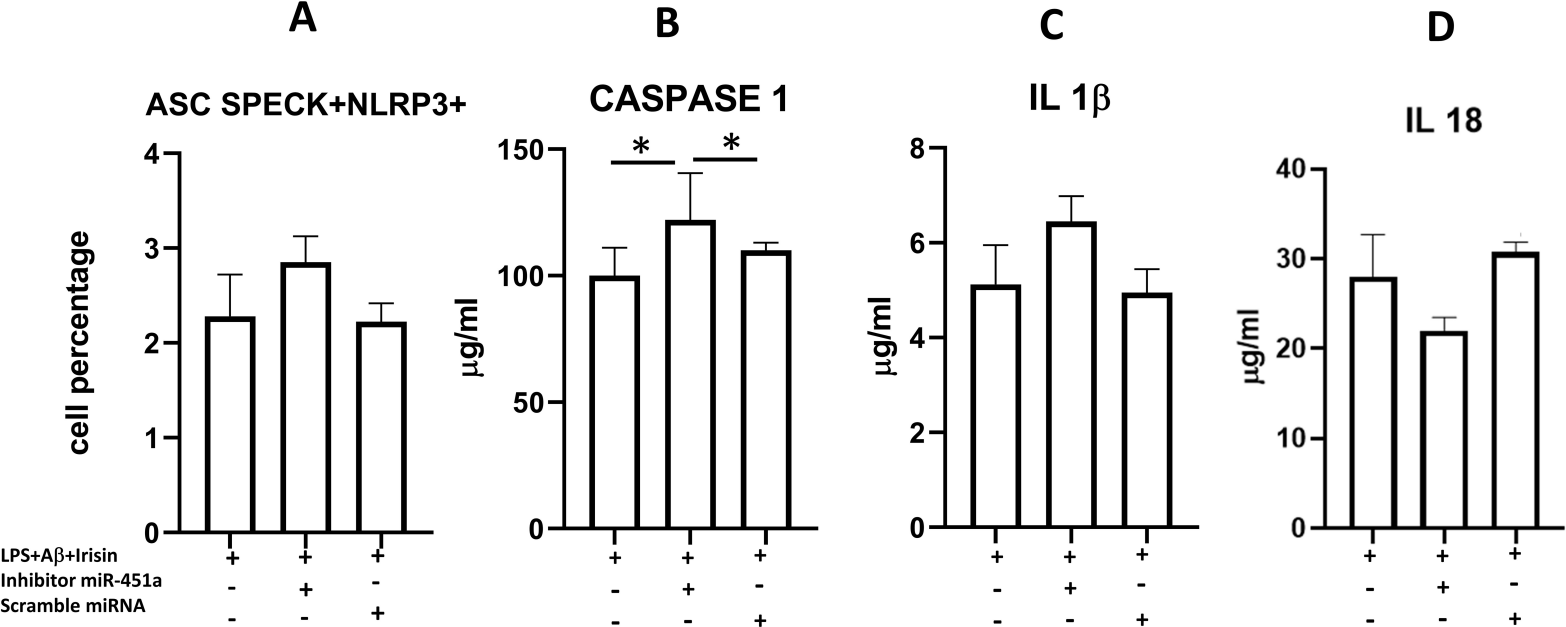
Summary of percentage of the microglia showing intracellular NLRP3/ASC speck colocalization **(A)** and of activated caspase-1 release (**B**), IL-1β **(C)** and IL-18 **(D)** production in supernatants of untransfected (MED) or transfected with miR-451a inhibitor or scrambled miRNA hTERT microglia. Cells were LPS-primed and Aβ_1-42_- stimulated in presence of irisin. Data are presented as median and interquartile range from n=3 independent experiments. Statistical significance is indicated: *p<0.05.

As shown above, intracellular NLRP3 colocalization with ASC speck was significantly reduced in hTERT cells that were LPS-primed and Aβ_42_ -stimulated in the presence of irisin, which was partially abolished by miR-451a silencing, although this difference was not statistically significant (**Figure 8A**). Notably, activated caspase-1 concentration was significantly increased in the supernatants of cells in which miR-451a was silenced compared to untransfected cells (p=0.006) and in those with scramble transfection (p=0.045) (**Figures 8B, C**). In contrast, IL-1β and IL-18 concentration didn’t reach statistical differences (**Figure 8D**).

## Discussion

The results of this study show that, in an *in vitro* model, irisin attenuates Aβ_1-42_- stimulated activation of microglia and is associated with modulation of miR-451a, and accompanied by downregulation of the expression of genes and proteins related to the NLRP3 inflammasome. The findings also showed that irisin-modulated changes in miR-451a were also associated with reduced TLR4 expression, suggesting a possible miRNA-mediated mechanism through which irisin may influence inflammation in human microglial model. This model is based on an acute inflammatory response which does not capture the chronic, progressive and multicellular features of AD. The model, nevertheless, might provide mechanistic insight into a potential anti-inflammatory pathway needing to be validated in more complex systems.

Aberrant activation of the NLRP3 inflammasome by Aβ and Tau has been implicated in chronic neuroinflammation, contributing to neuronal degeneration and cognitive decline in AD (7). Aβ aggregates activate microglia and astrocytes, promoting pro-inflammatory cytokines release (29), leading to neuronal loss, and cognitive impairment (6, 30). Chronic accumulation of Aβ leads to a dysfunctional microglial phenotype contributing to AD pathogenesis (31–33).

To explore aspects of microglial activation relevant to AD, we used a simplified and well-established LPS-primed human microglial cell model of NLRP3 inflammasome activation (30). LPS promotes transcription of inflammatory genes, including NLRP3 and IL-1β, via TLR4/NF-kB activation pathway, while Aβ_1–42_ peptide, the major component of AD senile plaques (34), serves as a second activation signal, triggering lysosomal damage, inflammasome assembly and the release of IL-1β and IL-18 (8).

Although NLRP3 inflammasome activation in AD is well established (4, 7, 30), the potential regulatory role of miRNAs and the capacity of irisin to modulate microglial function via miRNAs remain largely unexplored.

It has been suggested that irisin improves cognition in elderly individuals (17, 35), and was shown to counteract neuroinflammation in experimental models. Thus, in murine macrophages and microglia, irisin promotes the phenotypic switch from the pro-inflammatory M1 to the anti-inflammatory M2 state and attenuates NLRP3 inflammasome activation (16, 36). Consistent with these reports, our data showed that irisin reduced NLRP3 inflammasome activation, ASC speck formation, and pro-inflammatory cytokine production in Aβ_1-42_ -activated human microglia, supporting its anti-inflammatory effect in this model. Notably, while previous studies on irisin have mainly focused on activated cells, our findings showed that irisin alone does not modulate basal gene expression in unstimulated microglia, suggesting that its anti-inflammatory effects are primarily exerted through transcriptional regulation under pro-inflammatory conditions.

Based on these observations, we focused on miRNAs as potential modulators of irisin’s regulatory effects on NLRP3-mediated inflammation.

Several miRNAs have been identified as regulators of NLRP3 inflammasome activation, suggesting their potential role as therapeutic targets in AD. By modulating gene expression post-transcriptionally, microRNAs, together with other regulatory mechanisms, contribute to control of NLRP3 activation in microglia (37, 38). We focused on miR-223-3p, miR-7-5p and miR-451a, three microRNAs which are known to target the NLRP3-3’UTR and to play role in inflammasome regulation and inflammatory signaling; we reasoned that these three molecules could be the ones most likely to be involved in irisin-modulated pathways.

miR-223-3p is highly expressed in myeloid cells and regulates polarization and inflammation (39, 40). This miRNA modulates NLRP3 activation (25, 41) as well as many other key molecules involved in the NF-kB and MAPK pathways, including STAT3, IRAK-1, and RhoB (40). It is dysregulated in several neurodegenerative diseases (42–45), including AD.

miR-7-5p, is enriched in CNS and is involved in synaptic function and neuroprotection (46), reducing LPS-induced neuroinflammation (47). This miRNA targets NLRP3 (26), TLR4 (48, 49) and, notably, UBE2A, a ubiquitin enzyme involved in Aβ degradation in brain (50).

Finally, miR-451a, originally known for its roles in hematopoietic differentiation and tumor suppression, has recently emerged as a modulator of key immune signaling pathways, including NF-κB, AMPK, and PI3K, *via* its effects on NLRP3 (27) and TLR4 (22, 51). miR-451a has been reported to be involved in several inflammation-associated disorders (52) and is expressed in neurons and microglia. The expression of this molecule is reduced in microglia of an AD transgenic mouse model (APP/PS1) and in the cerebrospinal fluid of patients with AD, suggesting that it may play a role in the pathogenesis of AD (53).

In our model, irisin modulated the expression of all three miRNAs examined, supporting the notion that the NLRP3 inflammatory pathway is strongly regulated at the post-transcriptional level (37). LPS+Aβ_1–42_ stimulation downregulated miR-223-3p, leading to NLRP3 overexpression. Surprisingly, irisin further suppressed miR-223-3p expression in activated microglia, an unexpected finding, given the anti-inflammatory phenotype observed in our study. Nevertheless, it is important to emphasize that evidence on LPS-induced regulation of miR-223-3p in microglia is not fully consistent (54), with conflicting reports describing either up- (55) or down-regulation (56) of miR-223-3p expression.

Different patterns of miR-223-3p and miR-7-5p expression have been reported in different disease contexts, both of which are upregulated in the monocyte-derived macrophages (MDMs) of patients with AD (57), whereas they are downregulated in retinal microglia during degeneration (58), a condition associated with inflammation. These results indicated that the regulation of miR-223-3p (and its interplay with miR-7-5p) is likely to be highly cell- and context-dependent.

Irisin has been suggested to activate the JAK/STAT3 pathway in neurons and peripheral cells (14), although the evidence remains conflicting. Since STAT3 activation has been associated with miR-223-3p downregulation in other models (56) it is possible to hypothesize that irisin-induced activation of STAT3 contributes to the reduction of miR-223-3p expression in microglia. However, this hypothesis remains to be confirmed in activated microglia.

Conversely, irisin induced a significant and transient increase in miR-451a expression in the Aβ_1-42_-stimulated microglia. An inverse relationship was observed between miR-451a, TLR4, and NLRP3 expression in the presence of irisin, suggesting a potential association between irisin and regulation of their expression. These molecules may therefore contribute, at least in part, to the observed effects. In contrast, miR-7-5p upregulation was observed under both conditions but lacked a clear inverse relationship with these two targets. These findings suggest that miR-7-5p may be involved in additional or indirect pathways, or that its upregulation represents a compensatory response to strong miR-223-3p suppression induced by irisin, although the precise mechanisms warrant future investigation.

Based on these findings, we prioritized miR-451a among the three selected miRNAs for further mechanistic studies because its expression showed a clear irisin-induced modulation and an inverse relationship with key inflammatory targets (TLR4 and NLRP3), suggesting a potential contribution in mediating the observed anti-inflammatory effects.

Loss-of-function experiments were performed to determine whether inhibition of miR-451a reverses the effect of irisin on the TLR4/NF-kB/NLRP3 pathways, by assessing both genes and proteins expression, as well as NF-kB nuclear translocation.

The results confirmed that inhibition of miR-451a significantly increased TLR4 gene and protein expression in microglia, reversing the effect of irisin. These findings are in line with previous results obtained in a murine model (22, 51) and suggest that miR-451a contributes to the irisin-mediated modulation of NLRP3 inflammasome pathways in this model.

Inhibition of miR-451a only partially reversed NF-kB nuclear translocation and ASC speck colocalization; these findings suggest that the anti-inflammatory effects of irisin are not exclusively mediated by miR-451a but may also involve additional irisin-regulated miRNAs, such as miR-7-5p, or other signaling pathways. This may explain why inhibition of a single miRNA only partially counteracts the overall anti-inflammatory response. Further investigation will be needed to determine whether miR-451a directly regulates upstream adaptor molecules (such as MyD88) in microglial cells, as reported in neurons (51), or whether irisin acts through additional miRNAs or other signaling pathways. miR-451a gain-of-function experiments, which were not performed in the present study, could further clarify underlying molecular mechanisms.

miR-451a inhibition resulted in a significant increase in caspase-1 gene expression and activated protein release as well as an upregulation of IL-1β production in irisin-activated microglia. These results are consistent with a role of miR-451a in modulating TLR4 pathway. miR-451a inhibition resulted in decreased IL-18 production, indicating that the production of this cytokine is not likely to be TLR4-mediated. Notably IL-18 synthesis was suggested to be caspase 1-independent and to be associated with caspase 8 (59). Caspase-3 is involved as well in IL-18 activation; this caspase cleaves both pro-IL-18 and mature IL-18 at Asp71–Ser72 and Asp76–Asn77 to yield biologically inactive products (60). Thus, much of the regulation of IL-18 production may be post translationally driven by the enzymatic activity of members of the caspase family.

The early increase in miR-451a expression observed upon microglial stimulation in the presence of irisin may represent an immediate/early switch for TLR4 expression. This, in turn, may modulate downstream NF-kB signaling and NLRP3 inflammasome activity. These findings are consistent with previous reports describing the involvement of microRNAs in the rapid induction of immune response upon LPS stimulation (61, 62).

In summary, these results suggest that irisin is associated with modulation of microRNA expression in human microglia, with miR-451a a potentially contributing to the regulation of inflammasome-related pathways. The interactions among miR-451a, miR-7-5p, and miR-223-3p may reflect a complex and dynamic regulatory network that fine-tunes inflammasome activation in this *in vitro* model. However, while these findings provide mechanistic insight into irisin-associated signaling pathways, their relevance to neuroinflammatory processes *in vivo* and in disease contexts such as AD clearly remains to be established.

Some limitations must be acknowledged. This study employs an hTERT-immortalized human microglial cell line which likely differs phenotypically and functionally from primary microglia. In addition, only a limited number of candidate miRNAs was investigated, and the potential contribution of additional regulatory pathways was not explored. Validation in primary cells, induced pluripotent stem cells-derived microglia, and/or animal models is still lacking; further studies, including extended time-course analyzes in these models, will be necessary to confirm our preliminary findings. Importantly, no data are currently available on endogenous irisin dynamics in the brain, its ability to cross the blood-brain barrier under physiological or pathological conditions, or its *in vivo* functional relevance. Therefore, our findings should be interpreted as preliminary, hypothesis-generating insights into mechanisms potentially playing a role in the AD-associated neuroinflammation, but do not in any way can be described as being disease-related processes.

Nevertheless, this study also presents strengths, including the identification of a miRNA-associated mechanism underlying the anti-inflammatory activity of irisin in this *in vitro* model and the comprehensive characterization of the NLRP3 pathway via gene expression, protein levels, inflammasome assembly, and cytokine production.

In conclusion, these findings indicate that irisin is associated with anti-inflammatory effect in human microglia, through a coordinated modulation of microRNAs, with miR-451a as a potential contributor to the regulation of TLR4/NF-kB/NLRP3 inflammasome pathways. This *in vitro* model suggests a possible mechanism through which irisin may influence microglia inflammatory response. Further preclinical and clinical studies are required to assess the relevance of these findings to neuroinflammatory processes and, possibly, to AD pathophysiology.

## Data Availability

The raw data supporting the conclusions of this article will be made available by the authors, without undue reservation.
