# Supplementary material for: Irisin hampers β-amyloid-induced microglial inflammation via the miR-451a/TLR4/NLRP3 axis
Source: Front Immunol. 2026 Apr 23;17:1761520. doi: 10.3389/fimmu.2026.1761520 (PMC13149183; doi:10.3389/fimmu.2026.1761520)

Irisin hampers β-amyloid-induced microglial inflammation *via* the miR-451a/TLR4/ NLRP3 axis

**Roberta Mancuso^1^, Marina Saresella^1^, Riccardo Nuzzi^1^, Simone Agostini^1*^, Ivana Marventano^1^, Ambra Hernis^1^, Francesca La Rosa^1^, Federica Piancone^1^, Mario Clerici^1,2^**

^1^IRCCS Fondazione Don Carlo Gnocchi, Milan, Italy.

^2^Department of Pathophysiology and Transplantation, University of Milan, Milan, Italy.

Supplementary Material

**Supplementary Table 1**: List of genes probe assays and miRNAs primers used for quantification by droplet digital PCR (ddPCR).

| Target | Species | Fluorophore | Assay ID | Supplier |
| --- | --- | --- | --- | --- |
| NLRP3 | Human | FAM | dHsaCPE5058640 | Bio-Rad® |
| CASP1 | Human | FAM | dHsaCPE5031512 | Bio-Rad® |
| IL-18 | Human | HEX | dHsaCPE5038347 | Bio-Rad® |
| PYCARD | Human | FAM | dHsaCPE5033926 | Bio-Rad® |
| IL-1β | Human | HEX | dHsaCPE5057883 | Bio-Rad® |
| TLR2 | Human | FAM | Hs00152932 | ThermoFisher® |
| TLR4 | Human | VIC | Hs00152939 | ThermoFisher® |
| miR‑451a | Human | - | YP02119305 | Qiagen® |
| miR‑7‑1‑5p | Human | - | YP00205877 | Qiagen® |
| miR‑223‑3p | Human | - | YP00205986 | Qiagen® |

**Supplementary Figure 1.** hTERT microglial cells were left unstimulated (MED) or treated with irisin. mRNA expression of inflammasome-related genes (A, **NLRP3**; B, **PYCARD**; C, **caspase-1**; D, **IL-1β**; E, **IL-18**) was quantified by ddPCR. Data are expressed as copies/ng of RNA and presented as mean ± standard deviation (SD) from n = 3 independent experiments.


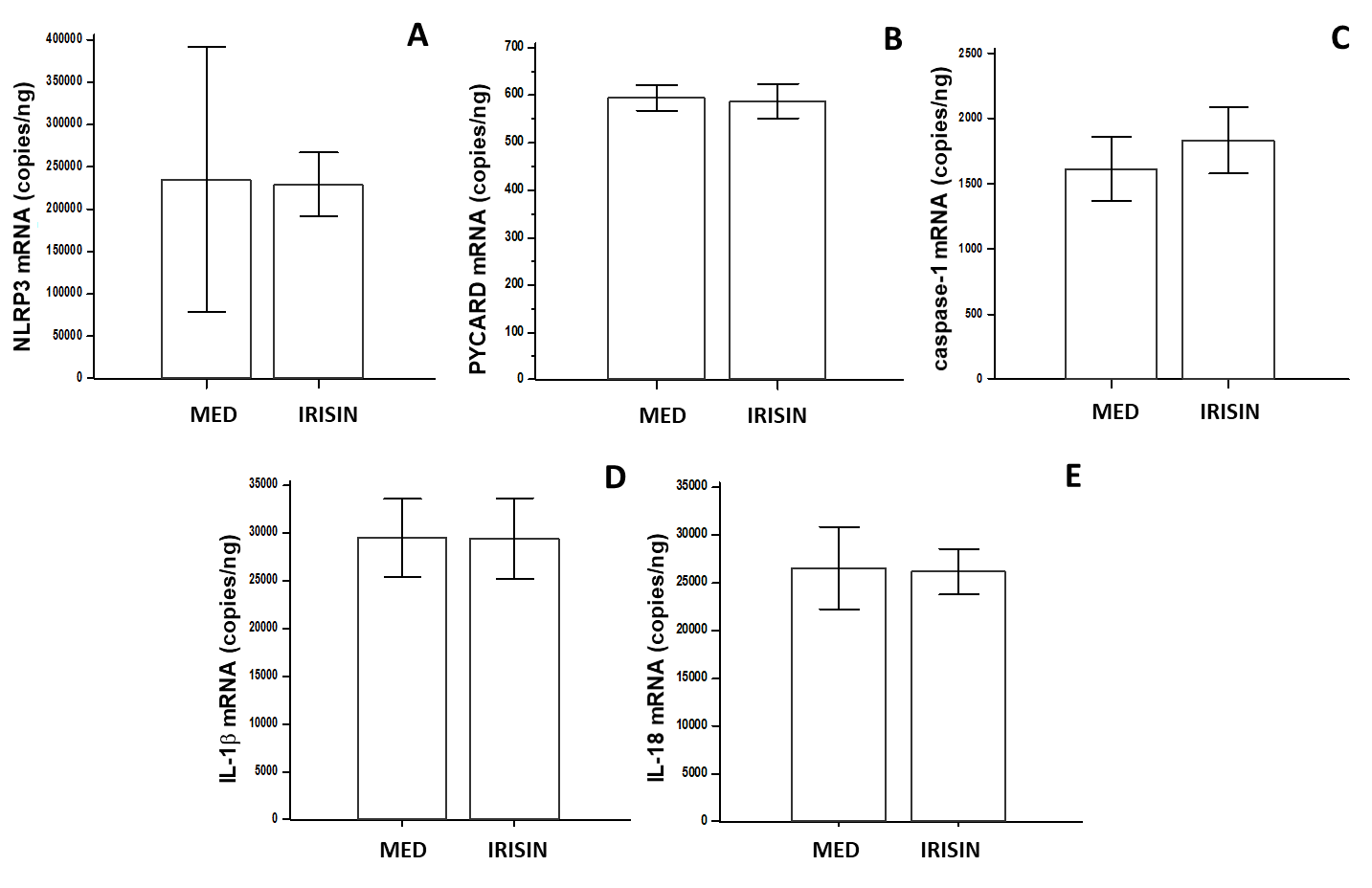

Supplement: Supplementary file 2 [file Table1.docx]
